# Understanding Stress Response in Superorganisms: A Multi‐Level and Cybernetic Approach

**DOI:** 10.1002/ece3.72626

**Published:** 2025-12-09

**Authors:** Fabio Sgolastra

**Affiliations:** ^1^ Dipartimento di Scienze e Tecnologie Agro‐Alimentari Alma Mater Studiorum Università di Bologna Bologna Italy

**Keywords:** biological organization, collapse, complexity, eusociality, stressors

## Abstract

This review aims to analyze the current knowledge and propose new approaches to understand how environmental stressors impact insect superorganisms across different levels of biological organization. In all multicellular organisms, stress‐response mechanisms are hierarchically organized, with processes at lower levels (e.g., cellular) nested within higher levels (e.g., tissues, organs, etc.). Understanding how these responses scale across organizational levels is essential for predicting stressor effects at the organism or superorganism level. Superorganisms, such as honeybee and ant colonies, represent good models to study these dynamics, because they present an additional level of biological organization (e.g., colony level) compared to other pluricellular organisms. This added level of “organismality” allows researchers to observe, at a macroscopic scale (individuals within a colony), mechanisms analogous to those operating at the microscopic scale (cells within an organism). Cybernetic principles—focused on feedback, regulation, and networked interactions—provide a powerful framework for analyzing how the networked structure of the superorganism responds to stress. The final goal is to identify common features across different levels of biological organization that may explain stress response in complex systems.

## Graphical abstract

All multicellular organisms have a nested stress response system, and understanding how stress effects propagate across biological levels is key to predicting their impact on the organism or superorganism. Superorganisms, like honeybee and ant colonies, represent good models to study stress response systems across different levels of biological organization. In this review I analyze the current knowledge and propose a cybernetic approach to understand the mechanisms that mitigate or exacerbate the impact of environmental stressors across different levels of biological organization in insect superorganisms.

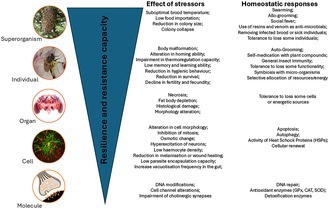

## 1 Introduction

A superorganism is defined as a group of genetically related individuals functioning as a collective unit. From an evolutionary perspective, superorganisms represent the highest level of biological organization, encompassing species, such as honeybees, wasps, ants and termites, with advanced eusociality characterized by cooperative brood care, reproductive division of labor, and generational overlap (Wilson and Hölldobler 2005). One possible theory to explain the evolution of eusociality is that the emergence of this level of organismality represents an adaptive response to environmental stressors (biotic and abiotic) operating across multiple levels of biological organization (Wilson and Hölldobler 2005). Understanding the interaction between superorganisms and stressors—particularly how this interaction scales across different levels of biological organization—is essential not only for uncovering the evolutionary mechanisms underlying the origin of superorganisms, but also for practical applications, including the development of effective management strategies for insect conservation (i.e., bees as crop pollinators) and pest control (i.e., invasive species of ants and termites in urban areas). In this review, I propose to integrate experimental and computational methods and to apply the principles of cybernetics, a discipline that studies systems characterized by self‐regulation, feedback loops, and network dynamics, to explain the fundamental mechanisms that govern superorganism responses to stressors (Stebbing 2011). Mechanisms that operate at various levels of biological organization and may have evolved as adaptations to cope with environmental stressors (Steinberg 2012).

In principle, all organisms are adapted to environmental conditions under which their survival and reproductive capacities are optimized. However, when organisms move outside of these optimal conditions, they enter a stress zone. The physiologist Hans Selye defined “*stressor*” as any factor, externally and internally, that produces an alteration of the organism's homeostasis, that is, its ability to maintain optimal internal conditions (Selye 1956). More specifically, environmental stressors are climatic, chemical and biological factors that, either in isolation or in combination, significantly impair organism performance or functions, pushing it into the stress zone where survival and reproductive capacity are affected (van Straalen 2003). According to Steinberg (2012), the stress zone and its associated response at the (sub‐)individual level can be divided into three distinct phases: alarm, resistance, and exhaustion. The alarm phase involves biochemical and genetic changes, such as the upregulation of stress‐response genes and the activation of antioxidant enzymes, that occur without impacting the organism's vitality, growth, or reproduction. During the resistance phase, defense mechanisms are activated—such as protein repair pathways and biotransformation processes—and are accompanied by the first signs of reduced physiological performance. The exhaustion phase is marked by the failure of essential cellular functions, resulting in chronic damage and, ultimately, death (Steinberg 2012).

Organisms may temporally maintain survival and reproductive capacity under suboptimal, stressful conditions, through tolerance and mitigation strategies. In biology, *tolerance* is the ability of an organism to endure a stressor without necessarily reducing its presence or severity (Elizabeth and Robert 2015). In such cases, the organism's performance is maintained despite the stress. Mitigation, on the other hand, involves active responses aimed at reducing or avoiding the stressor. These responses may be behavioral, physiological, or immune‐based, and are intended to minimize either the presence or the impact of the stressor. Moreover, the stress response is not always maladaptive; in some cases, it can have beneficial effects, such as in the phenomenon of hormesis, where low doses of a toxicant trigger adaptive and beneficial responses. According to Calabrese and Baldwin (2001), hormesis acts as a compensatory mechanism in response to disrupted homeostasis. Another positive effect is the cross‐tolerance that typically occurs when exposure to one stressor may result in efficient resistance to other stressors (Steinberg 2012).

Depending on the level of exposure, both in terms of duration and intensity, stressors can impact various levels of biological organization, impairing the function of cells, tissues, organs, individuals, and, in the case of superorganisms, entire colonies (Sulmon et al. 2015). For example, in honeybees ( *Apis mellifera* ), it has been demonstrated that exposure to neurotoxic pesticides leads to brain cell apoptosis, which has a detrimental effect on the nervous system, thereby reducing the learning and memory capacity of adult bees (Chen et al. 2023). In the carpenter ant *Camponotus fellah* , social isolation induces changes in the expression of oxidoreductase genes and the accumulation of reactive oxygen species (ROS) leading to a reduction in adult life span (Koto et al. 2023). Effects observed at one level of biological organization can propagate to the higher levels as the intensity or duration of exposure increases. However, our understanding of how stressors interact with superorganisms becomes gradually more complicated scaling up from the lower to the higher level of biological organization due to the increase in complexity of environmental and biological interactions (Sulmon et al. 2015). In fact, although numerous studies have elucidated the mechanisms by which superorganisms tolerate biotic stressors (e.g., pathogens and parasites) (e.g., Cremer et al. 2007) and identified pathways leading to superorganism collapse (e.g., Barron 2015), the scaling of stress responses from subcellular to superorganismal levels remains poorly understood. This context raises several specific questions: What degree of perturbation can be buffered by a superorganism? To what extent can a superorganism tolerate environmental stressors before collapsing, and what factors influence the trajectory of its recovery following disturbance? What are the key dynamics and mechanisms across different levels of biological organization that determine a superorganism's capacity—or lack thereof—to withstand stressors? Can the impact of stressors at the colony level be predicted by assessing effects at lower levels of biological organization? Can effects observed at lower levels of biological organization serve as early warning indicators for the collapse of superorganisms? What role have stress responses played in the evolution of superorganisms?

To address these questions, it is essential to adopt a holistic or systems‐based approach that emphasizes the interactions among components rather than viewing them in isolation. Within this framework, the effects of stressors at various biological levels are understood as part of a broader, interconnected network, providing a more integrated perspective on the functioning and resilience of complex biological systems (Kohl et al. 2010; Scheffer et al. 2018).

Drawing primarily from insights on the superorganism, the honeybee ( *Apis mellifera* ) colony—the most extensively studied and best‐documented social insect in the literature—but see: Bordoni et al. (2017); Parr and Bishop (2022) Svoboda et al. (2023), this review provides an overview of the current understanding of how superorganisms respond to environmental stressors across different levels of biological organization. It also offers methodological strategies and future perspectives to predict the mechanisms underlying superorganism collapse.

The review is organized into five sections, following a general introduction. In the first section of the review, the evolution of superorganisms as a new level of biological organization is discussed. The second section examines how these mitigation mechanisms can be disrupted by environmental stressors, leading to colony collapse. In the third section, an integrative approach that combines experimental and computational models is proposed to enhance our understanding of how stressor effects scale from lower to higher levels of biological organization. This approach seeks to identify and elucidate the underlying mechanisms operating at lower organizational levels that are ecologically relevant to superorganism performance. In the fourth section, the possibility of applying the cybernetic approach in understanding colony performance is discussed. In the last section of the review the possibility of extending this approach to other complex systems is proposed with the aim of shedding light on broader biological questions, such as the evolution of eusociality and the origins of multicellularity.

## 2 The Superorganism: A New Level of Organismality in Response to Stressors

The concept of superorganism was first proposed by William Morton Wheeler (1865–1937). He described social insect colonies as “superorganisms”, whose individual members appear to operate as a single unit (Wheeler 1911). More precisely he found a “striking analogy between the ant colony and the cell colony which constitutes the body of a Metazoan animal; and many of the laws that control the cellular origin, development, growth, reproduction and decay of the individual Metazoan, are seen to hold good also of the ant society regarded as an individual of a higher order. As in the case of the individual animal, no further purpose of the colony can be detected than that of maintaining itself in the face of a constantly changing environment till it is able to reproduce other colonies of a like constitution. The queen mother of the ant colony displays the generalized potentialities of all the individuals, just as the Metazoan egg contains *in potentia* all the other cells of the body. And, continuing the analogy, we may say that since the different castes of the ant colony are morphologically specialized for the performance of different functions, they are truly comparable with the differentiated tissues of the Metazoan body.” (Wheeler 1910).

Eusocial insects, often referred to as the “truly” social insects, include all ant and termite species, as well as several species of bees and wasps. These organisms exhibit three key characteristics: (1) cooperative brood care among individuals of the same species; (2) reproductive division of labor, whereby reproduction is restricted to one or a few individuals while others—typically sterile or facultatively sterile—contribute to colony maintenance and care; and (3) generational overlap within the colony, allowing offspring to assist in the care and provisioning of siblings and to participate in the overall functioning of the colony. The division of labor among sterile castes is regulated by a combination of genetic and pheromonal mechanisms that optimize internal nest function. These individuals perform a range of tasks, including thermoregulation, nest defense, foraging, and brood rearing (Wilson and Hölldobler 2005).

Although eusocial insects comprise only ca. 2% of the ca. 900,000 insect species known globally, these species represent more than half of the global insect biomass (Rosenberg et al. 2023; Starr 2021). Eusocial species are present only in 15 out of 2600 living taxonomic families of insects but their origin was an important step in the evolution of life on our planet and represents a successful strategy that provides an additional level of resilience to environmental stressors (Wilson and Hölldobler 2005).

The ecological advantages of eusociality over solitary life strategies are apparent in the capacity of eusocial insects to exploit trophic resources across extensive areas and to rear thousands of larvae simultaneously. These capabilities confer substantial adaptive benefits, enabling the construction of complex nests with enhanced defensive features and improved regulation of the internal microclimate. From an ecological standpoint, eusocial insects often dominate the central, more stable regions of terrestrial ecosystems, whereas solitary species are typically confined to more peripheral or transient ecological niches (Wilson 1990).

The evolutionary origins of insect sociality, particularly eusociality, remain a central question in evolutionary biology. Kin selection theory, grounded in the concept of inclusive fitness—whereby individuals increase their genetic representation by enhancing the reproductive success of relatives—has long been the dominant explanatory model. High genetic relatedness within colonies has been cited as evidence for kin selection's key role in the evolution of eusociality. However, Nowak et al. (2010) argue that relatedness is more likely a consequence than a cause of eusocial evolution. In their view, eusociality arises through a series of pre‐adaptive traits shaped by natural selection, initially at the individual level and subsequently through group‐level processes. These traits confer significant ecological advantages over solitary individuals or less cooperative groups, particularly in competition for nesting sites, resource acquisition, and resilience to environmental stressors—favoring group selection. Thus, it can be expected that the stronger the selective pressures at the group level, the more cohesive the group members will be. In line with this view, Wheeler proposed that an insect colony functions as a unified living organism, maintaining its structural and functional integrity in response to stressors. He introduced the concept of “social homeostasis” to describe the integrated physiological and behavioral mechanisms through which colonies regulate internal conditions to optimize development and reproduction (Wheeler 1928).

**FIGURE 1:**
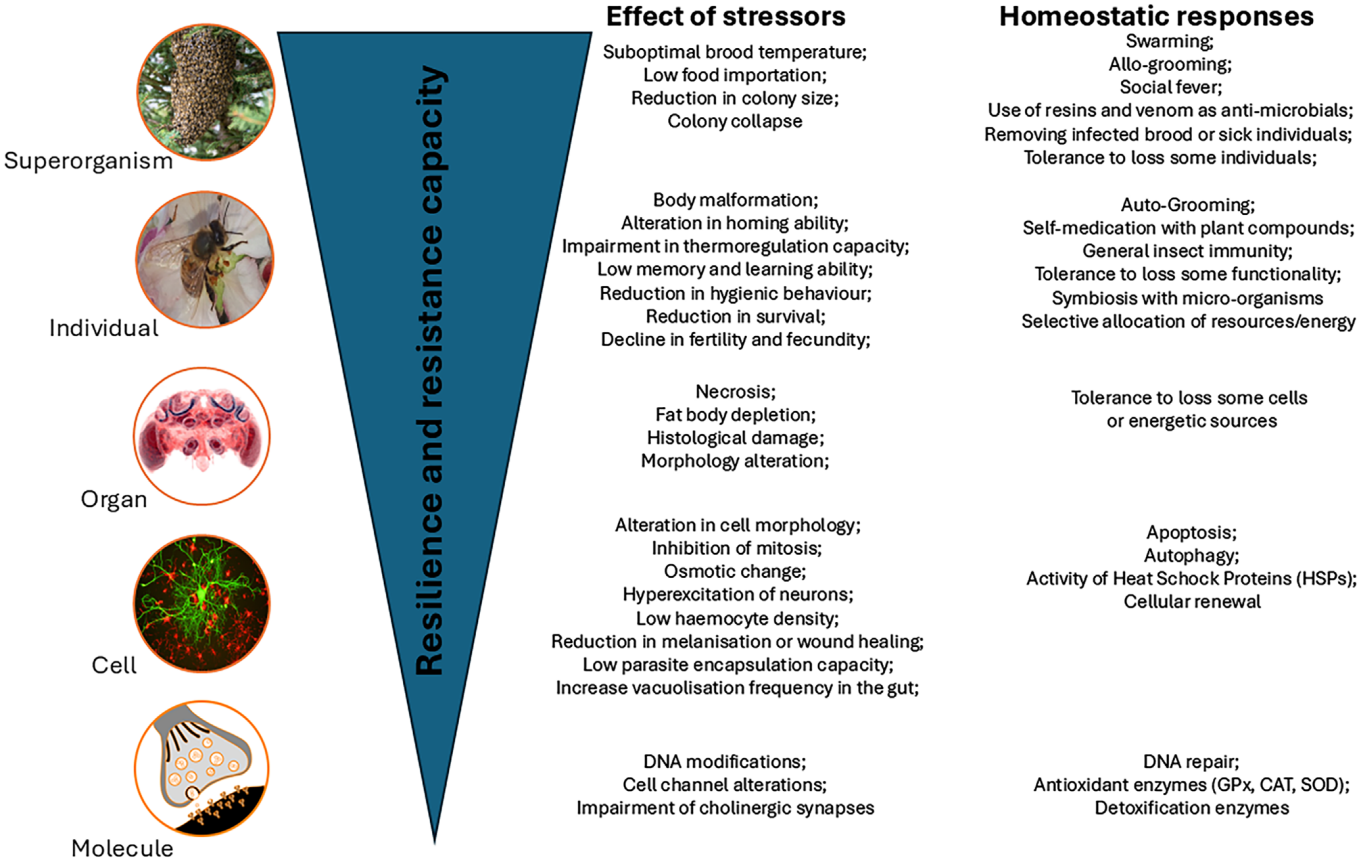
Potential effects of environmental stressors and homeostatic responses across levels of biological organization in a superorganism. The central hypothesis is that resilience and resistance capacities increase from lower to higher levels of biological organization, as each successive level is capable of buffering minor perturbations occurring at the preceding level.

According to Steinberg (2012), stressors have been an important ecological driving force in the evolution of life, and Metazoan animals have evolved several strategies to tolerate or mitigate, at least temporally, stress conditions that can be observed at different levels of biological organization, from genes and cells to individuals (Steinberg 2012). As shown in Figure 1, the stress response system is hierarchically organized like a set of Chinese boxes, where lower‐scale mechanisms are embedded within higher‐level ones, resulting in progressively greater protection across levels of biological organization. Yet, eusocial insects have evolved an additional adaptation to face abiotic and biotic stressors (Figure 1) (Pull and McMahon 2020). This adaptation involves at least two general strategies that partially overlap, referred to as “superorganism resilience” (Straub et al. 2015) and “social stress protection” (Cremer and Pull 2024). The former is defined as the ability to tolerate the loss of somatic cells (= workers) as long as the germ line (= reproduction) is maintained (Straub et al. 2015). The second one includes homeostatic mechanisms and unequal distribution of stress exposure among the colony members, that are able to buffer the negative consequences of extreme stressors on many members of social insect colonies (Walton et al. 2024). The “social immunity”, which includes the collective defenses of social insects against parasites and pathogens (Cremer and Pull 2024), is an important part of the “social stress protection” and, like the immune system in pluricellular organisms, has evolved in superorganism to increase the fitness of the colony (Pull and McMahon 2020). In this context, the evolution of cooperation—whether among cells within pluricellular organisms or among individuals within superorganisms—can be interpreted as an adaptive strategy against pathogenic threats. In species exhibiting parental care and possessing specific pre‐adaptations, such as pathogen detection, grooming behaviors, and the use of antimicrobial substances, group defense mechanisms may have evolved and been maintained through inclusive fitness benefits, thereby enhancing group survival. These pre‐adaptive traits may have emerged prior to—and thus facilitated—the transition toward superorganismality. Upon reaching this higher level of biological organization, natural selection begins to act at the level of the group, thereby promoting the evolution of more specialized forms of social immunity, including behaviors such as “social fever” (e.g., a collective defense strategy in honeybees characterized by the increase of the nest temperature to fight heat‐sensitive pathogens) and the removal of infected individuals (Figure 1) (Pull and McMahon 2020). Collectively, these lines of evidence support the hypothesis that an enhanced capacity to cope with environmental stressors was an important driving force in the evolution of eusociality.

## 3 Stability and Collapse: Two Sides of the Same Coin

A system is considered stable when it maintains its reference condition (state or dynamics), and consequently preserves its function, structure, and identity despite changing conditions (Van Meerbeek et al. 2021). Two key components of stability that are particularly relevant when examining the impacts of environmental stressors in ecology are the concepts of resilience and resistance. More specifically, *stress resistance* is the capacity to prevent stress from crossing the *tipping point*—the critical threshold in a complex system at which a small perturbation can trigger a significant and often irreversible shift or outcome—thereby avoiding maladaptive effects (Latty and Dakos 2019). In contrast, *stress resilience* is the capacity to recover appropriate function in response to a stressor. Both are under the umbrella of adaptation to stress (Miller et al. 2017) that aims to maintain or restore the system's stability or homeostasis—that is, its ability to maintain specific functions in the face of change (Baggio et al. 2015). Because collapse occurs when the system loses its stability, collapse and stability can be considered two sides of the same coin. However, the transition between these two “states” may involve varying degrees of instability, during which the system can still eventually recover. According to Scheffer et al. (2018), the transition from a stable, “resistant” or “resilient” system, to an unstable, “weak” one approaching the tipping point, is marked by a slower recovery rate following small perturbations. However, when the tipping point is reached the collapse happens inevitably. Due to the complex network‐like structure of the (super)organisms characterized by interconnected subsystems (e.g., genes, cells, organs), the resilience of the whole depends on the resilience of subsystems or, in other terms, the collapse of the entire system results from how each individual subsystem responds to perturbations or stressors. Superorganisms have evolved multiple layers of mechanisms against stressors to prevent collapse (Figure 1), and as a result of cumulative homeostatic responses across levels of biological organization, resilience and resistance are expected to increase from lower to higher levels of biological complexity (Latty and Dakos 2019; Crall and Raine 2023). These homeostatic mechanisms protect organisms (or superorganisms) from dramatic changes and are regulated by feedback loops that return the system back to a set point or threshold. At lower levels of biological organization (e.g., subcellular or cellular), for instance, environmental stressors can cause DNA damage, alterations in lipid and protein metabolism and downregulation of P450 gene expression linked to detoxification and immunity (Kültz 2005). To mitigate stressors, cells activate several specific pathways that include heat shock and oxidative stress responses, DNA repairs and several detoxification mechanisms. In *extrema ratio*, these actions can result in cell death by apoptosis, autophagy or necrosis that may mitigate damages at higher levels of biological organization (e.g., by removing damaged cells to prevent organ dysfunction) (Desneux et al. 2007; Tosi et al. 2022) (Figure 1). These cellular stress responses are evolutionarily conserved and shared across a wide range of organisms, from yeast to humans. Nevertheless, the responses at higher levels of biological organization can exhibit greater variability due to the increased level of complexity (Kültz 2005). At the individual level, environmental stressors can, for example, produce malformations during development, alter the normal behaviors (e.g., lower learning and memory capacity, disrupted navigation ability) and reduce longevity and fecundity (Desneux et al. 2007; Tosi et al. 2022). In response to these effects, organisms can react by selective allocation of resources toward self‐maintenance and reproduction and may activate several physiological and behavioral mechanisms, such as self‐medication, auto‐grooming, and processes linked with general insect immunity (e.g., melanization, AMPs) (Figure 1). At the superorganism level, stressors can alter thermoregulation capacity, reduce colony size, and eventually lead to collapse (Ulgezen et al. 2021). However, superorganisms can mitigate stressors with several preventive and curative mechanisms such as, allo‐grooming, hygienic removal of dead or dying individuals, use of antimicrobial plant resins and venom, and self‐removal of sick individuals (altruistic suicide) (Scharf et al. 2012; Easton‐Calabria et al. 2023) (Figure 1).

Despite these homeostatic responses, collapse can still occur as a result of the accumulation and cascading effects of numerous small perturbations across different levels of biological organization (Latty and Dakos 2019). Moreover, because the stress response is energetically costly across all levels of biological organization, the energy expended to cope with a given stressor may leave fewer resources to face the subsequent stress exposures. Several studies conducted with the honeybee *A. mellifera* have shown that bees infected with viruses or other pathogens are more sensitive to pesticides and vice versa by disrupting immune response and energy metabolism (i.e., Di Prisco et al. 2013; Doublet et al. 2014; Tadei et al. 2025). Similar pesticide‐parasite combination effects have been observed in workers of the black garden ant, *Lasius niger* (Schläppi et al. 2021).

At the level of superorganism, the effects of stressors can be even more complex and difficult to predict. In fact, the impact of a stressor at any given level is influenced by the response of lower levels, and due to the complex, interconnected relationships among biological components (organelles, cells, tissues, individuals), elements not directly exposed to the stressor may also be affected. For example, the disruptions in some organ functions caused by pathogens or pesticides, although not lethal at the individual level, can strongly influence colony health by interfering with nest homeostasis and social regulation. In honeybees, nurse bees exposed to neonicotinoid pesticides show smaller hypopharyngeal glands and a reduction in royal jelly production, the main larval food synthesized by these glands, with an adverse effect on brood development (Wessler et al. 2016). Although the colony shows short‐term resilience by increasing the brood initiation rate to compensate for elevated brood mortality, this compensatory mechanism ultimately reduces colony survival over the long term (Schott et al. 2021). Consequently, the functionality of the hypopharyngeal glands holds greater significance within the social dynamics of the colony than for the survival of an individual nurse bee, exemplifying the unique challenges associated with assessing stressors in superorganisms like honeybee colonies (Berenbaum and Liao 2019).

Understanding how the impact of stressors scales up from lower to higher levels remains an unresolved question and predicting when and why superorganism collapse occurs remains a significant challenge, largely due to the complex interplay between stressor effects at the individual level and their emergent consequences at the colony level (Crall and Raine 2023). The eradication of ant and termite colonies for pest control (Box 1), along with the phenomenon of honeybee colony collapse (discussed in the following paragraph), provides two real‐world examples illustrating the complexity of superorganism failure.

BOX 1 **How to kill a superorganism: the use of toxic bait to eliminate ant and termite colonies.**

Ants and termites play a crucial ecological role in many ecosystems, contributing to seed dispersal, scavenging, and soil aeration. However to humans, in highly anthropogenic environments, they can also become significant pests. Their social organization with thousands of individuals and their strong adaptation capacity to different environments makes their control extremely difficult. Many pest management methods have been developed to reduce their impact on human life and, among them, toxic bait is currently one of the main management tools. Baiting is based on the use of attractive insecticide‐treated food which is actively collected and taken back to the nest by foraging workers. Inside the nest, baits are transferred throughout trophallaxis to other individual workers, brood, and the queen, potentially leading to colony collapse. To be effective, the toxicant used in the bait should be non‐repellent and have delayed action. Foragers should be able to return to the nest and pass the toxicant to members of the colony. In addition, the toxicant should be effective over an extended dose range so that the residual toxin is still able to kill or affect the members of the colony even after its dilution during the spread of the toxicant throughout the nest. Mechanisms behind the activity of toxic baits in killing the colony are often complex and subtle. These mechanisms were demonstrated in a toxic bait for leaf‐cutting ants by Gandra et al. (2016). The authors showed that the colony suppression was linked to the effects of fipronil, the insecticide used in the bait, on forager nestmate interactions (auto‐ and allogrooming) and waste removal and, overall, on the capacity of minor workers in fungus garden cultivation and progeny handling. Indeed, fipronil did not interfere with bait pick‐up by the foraging workers, who were able to introduce the bait into the nest, and the queen's reproduction capacity, but, rather, with the waste removal activities of minor workers within the nest. The impairment of protective and hygienic behaviors, led to a fast decay of the fungus garden and likely progeny mishandling, ultimately leading to colony collapse within 8 days. The decline of the fungus garden, which became heavily contaminated with a virulent antagonist fungus of ant fungus, was primarily due to the effects of fipronil on the hygienic behavior of minor workers. Later the colony suppression was further enhanced by the interaction of the neurotoxic insecticide with the pathogen proliferation. The authors conclude that combination with sub‐lethal doses of neurotoxic insecticides and pathogens can result in a promising control strategy against ants (Santos et al. 2007).

**FIGURE 2:**
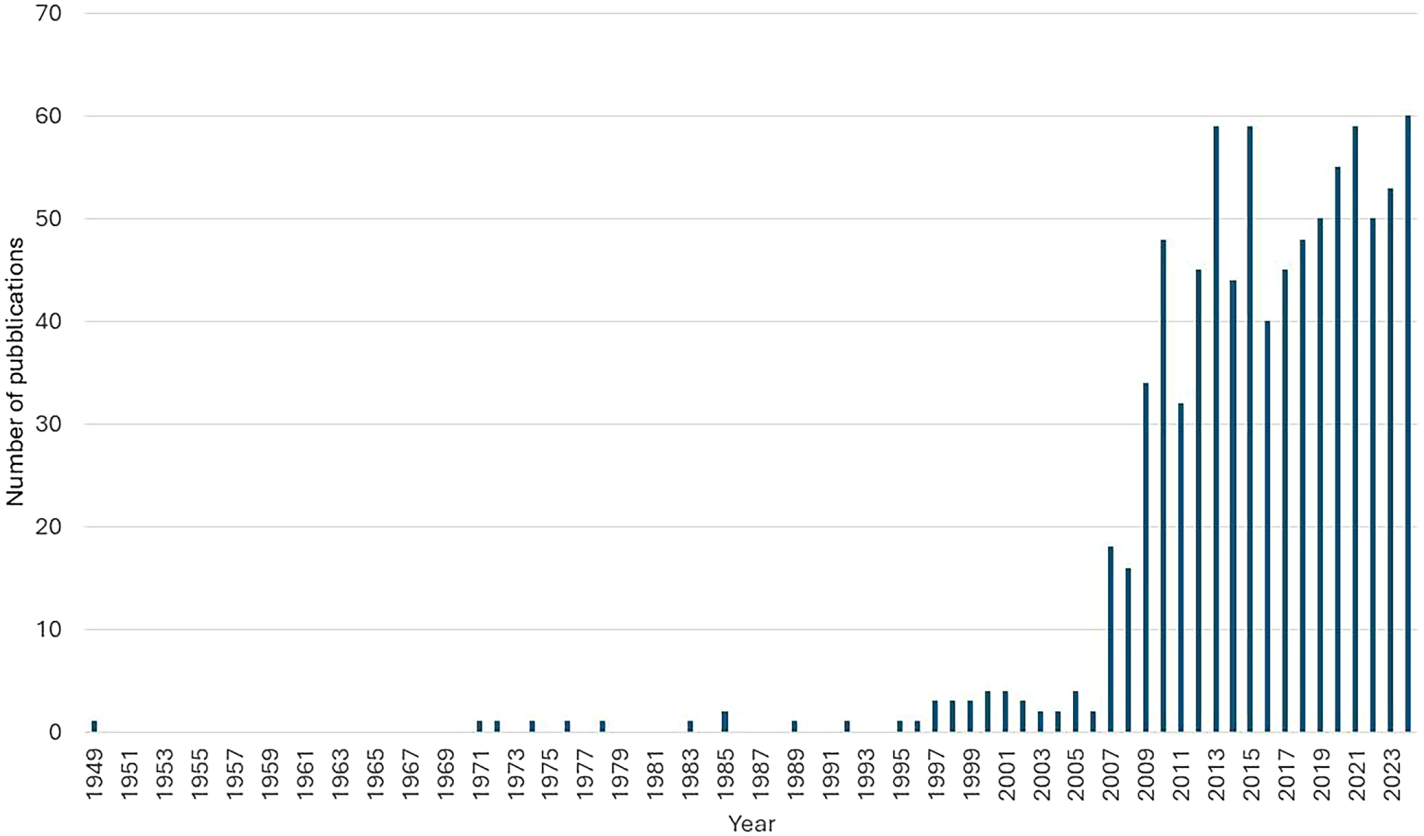
Number of scientific publications per year in Scopus using the key words: “Bees” AND “Collapse” (Search date: 13/01/2024).

**FIGURE 3:**
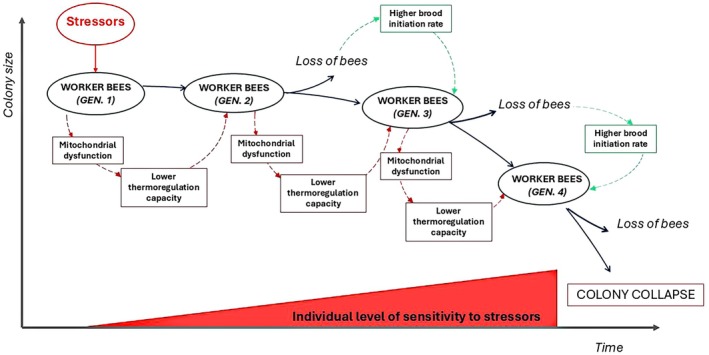
Conceptual model of colony collapse. Stressors, whether acting individually or synergistically, impact worker bees in the first generation and indirectly affect subsequent generations through reinforcing feedback loops (in red). Workers reared under suboptimal temperatures exhibit mitochondrial dysfunction (Lu et al. 2020) and reduced thermoregulation capacity, impairing the performance of future adult bees. These effects intensify over time in a cascading way, as increased individual sensitivity to additional stressors (e.g., pesticides; Medrzycki et al. 2010) further exacerbates colony decline. The loss of bees in each generation can only be partially mitigated by compensatory mechanisms such as increased brood initiation rates (feedback loop shown in green).

Honeybee colony collapse has been the subject of substantial research since 2007, when the phenomenon of “Colony Collapse Disorder” (CCD) was first identified in the United States (Figure 2). CCD is characterized by the rapid depopulation of colonies, wherein worker bees seemingly vanish from the hive, leaving behind the queen, brood, and food stores, without any overt signs of disease (vanEngelsdorp et al. 2009). Yet, CCD is only one of the possible causes of the increasing colony mortality rate observed recently around the world (Vanengelsdorp and Meixner 2010). Currently, scientists agree that most of the colony mortalities arise from complex interactions in which the sublethal effects of multiple stressors on individual bees are magnified at the colony level, ultimately resulting in colony collapse (Barron 2015). However, biological mechanisms driving the processes underlying colony failure remain largely hypothetical and require experimental validation (Barron 2015). One proposed mechanism involves the formation of feedback loops associated with inadequate brood incubation (i.e., chilling loops or repeated cycles of rearing under lower temperature conditions) (Oldroyd 2007; More et al. 2021). Thermoregulation in honeybee colonies represents a critical emergent property, as worker bees must maintain the brood area within a narrow range of temperature (ca. 34°C). Even slight deviations from this optimal range, caused by one or more stressors, can result in newly emerged adults with physiological and behavioral deficits (Oldroyd 2007). Workers reared at suboptimal (lower) temperatures may exhibit impaired homing abilities and reduced thermoregulation performance. As a result, they may rear the next generation of brood under suboptimal temperatures, potentially initiating a cascading effect that ultimately results in colony collapse (Figure 3). Although this hypothesis requires comprehensive experimental validation, partial evidence supporting it has been provided by both laboratory and field studies. Lu et al. (2012) showed that colonies exposed to neonicotinoid pesticides for 13 consecutive weeks exhibited normal development during summer and autumn but experienced a sudden decline and collapsed during winter. This phenomenon was linked to cumulative mitochondrial dysfunction across successive worker generations (Lu et al. 2020). Such sub‐individual effect, here defined as any effect observed at a level of biological organization lower than the individual, may impair the thermoregulation capacity of worker bees, potentially initiating the first chilling loops. Subsequent chilling loops may result from further thermoregulation deficiencies in workers reared under suboptimal temperature conditions. Partial experimental support for this explanation was provided by Medrzycki et al. (2010) that found a short longevity and a higher pesticide sensitivity in bees reared at suboptimal temperatures. These bees may exhibit higher sensitivity to additional stressors, undermining any potential mitigation measures implemented by the superorganism to prevent collapse, such as increasing the queen egg‐laying rate and other homeostatic responses reported in Figure 1. Another example of how effects at the individual level scale up to the colony level, ultimately leading to colony failure during winter, was proposed by Minaud Rebaudo, Davidson, et al. (2024) and Minaud, Rebaudo, Mainardi, et al. (2024). The authors hypothesized a cascade effect driven by a positive feedback loop, initiated when stressors reduce the longevity of winter bees. This reduction adversely impacts the cluster's size and cohesion, disrupting social thermoregulation and causing excessive consumption of honey reserves. The resulting depletion of honey reserves leads to starvation, further weakening the winter bees and reducing the colony population size until collapse ensues. The aforementioned examples show that when the colony size decreases below a certain number of individuals (e.g., the tipping point), colony collapse occurs quickly and in an irreversible way.

The conceptual model proposed by Minaud, Rebaudo, Davidson, et al. (2024) and Minaud, Rebaudo, Mainardi, et al. (2024) along with the framework illustrated in Figure 3 and the case study presented in Box 1, underscores that the collapse of a superorganism follows complex pathways, arising from intricate interactions among multiple factors or stressors (Barron 2015). This complexity is further exacerbated by the fact that these stressors in the field often act in combination rather than in isolation, potentially leading to synergistic or antagonistic interactions (Orr et al. 2020).

## 4 Studying Stressor Effects on Superorganisms: Integrating Experimental and Computational Research

As described in the previous section, the superorganism's response to stressors arises from complex interactions among multiple factors and processes, and the various constituent elements (e.g., molecules, cells, organs, and organisms) of the superorganism (Klein et al. 2017). Addressing this complexity while avoiding oversimplification requires an integrative approach combining empirical data (from laboratory and field experiments) and computational analysis. Surprisingly, few studies have investigated the impact of a single or multiple stressors by examining multiple endpoints (e.g., enzymatic activity, learning capacity, survival, etc.) across different levels of biological organization in superorganisms (but see: Chen et al. 2024; Miotelo et al. 2022). As a result, we lack understanding of how the effects detected at the molecular level might lead to irreversible alterations in physiology and behavior at the individual level and eventually result in colony collapse (Henry et al. 2015). An additional challenge lies in the fact that, while the effects of a stressor at the (sub‐)individual level can be readily studied under laboratory conditions, assessing its impact at the individual or colony level—if it is to be ecologically meaningful—requires observation under realistic field conditions. For this reason, it is essential to integrate laboratory experiments with field‐based studies. Laboratory studies, because they are conducted under strictly controlled conditions, are important to assess specific effects of single or a few stressors on (sub‐)individuals and to understand the basic mechanisms behind them. However, laboratory studies can hinder detection of connections between apparently isolated biological events, such as the interactions among multiple environmental variables and specific biological responses, and are not always feasible using full colonies (Sgolastra et al. 2020; but see: Pohl et al. 2025 for ants). Indeed, it is virtually impossible to replicate in laboratory conditions all potential combinations of stressors and environmental factors that may affect superorganism health. Furthermore, for some superorganisms such as honeybee colonies, due to their large foraging range and complex nest thermoregulation, cannot be maintained under confined conditions (e.g., laboratory or semi‐field setups) for extended periods. Nevertheless, field studies pose their own challenges, including a high number of confounding variables (e.g., climate variability, food sources availability, exposure to xenobiotics) and limited statistical power, as detecting significant effects often requires a large number of colonies (Cresswell 2011; Woodcock et al. 2016).

Computational research offers a viable approach to overcoming these limitations, bridging the gap between laboratory and field experiment outcomes. Several mathematical models have already demonstrated their utility in elucidating the processes underlying colony failure by linking the effects of stressors on individual bees to colony‐level dynamics (e.g., Khoury et al. 2011; Perry et al. 2015; Dennis and Kemp 2016; Bastiaansen et al. 2020; Bryden et al. 2013). Moreover, given the impracticality of experimentally testing all possible stressor combinations, models provide a valuable tool for exploring different scenarios and identifying the most relevant stressors influencing superorganism responses that can be tested experimentally. Finally, the outputs of these models can be integrated into more complex simulation models to inform decision‐making in areas such as environmental risk assessment and beekeeping management (EFSA 2016). Within this context, simulation models enhance our understanding of the relationship between individual responses to stressors and overall honeybee colony health and dynamics (More et al. 2021). However, to achieve accurate predictions, these models require extensive biological and environmental data derived from empirical studies. The integration of remote sensing data for large‐scale environmental monitoring (e.g., temperature, humidity, rainfall, colony size, in‐hive temperature) with Machine Learning and AI tools can also improve model accuracy, as proposed by Rafael Braga et al. (2020) for predicting the health status of honeybee colonies.

Modeling can also be useful to identify early warning indicators of colony collapse before reaching the *tipping point*. Using the simulation model BEEHAVE, Groeneveld et al. (2024) showed the number of capped brood to be a good early warning indicator of colony loss. Recently, Minaud, Rebaudo, Davidson, et al. (2024); Minaud, Rebaudo, Mainardi, et al. (2024); and Colin et al. (2024) have identified social thermoregulation as an early warning indicator of winter colony mortality that can be accurately and easily monitored by in‐hive temperature sensors. Because social thermoregulation and other colony functions are the result of the performance of single individuals, another possibility is to find a parameter or a set of parameters measurable at the (sub‐)individual level that can be useful to quantify the resilience of the whole colony. Alterations at the molecular, biochemical and cellular level due to stressors may be measured with several biochemical and physiological parameters, defined “biomarkers”, linked to metabolism, neurotoxicity, oxidative stress, genotoxicity, detoxification and immune systems. Biomarkers are potentially important diagnostic tools to quantify the status of the colony because they can be easily studied in laboratory experiments by analyzing individual insects (Caliani et al. 2021). These and other indicators at lower levels of biological organization (Minaud, Rebaudo, Davidson, et al. 2024; Minaud, Rebaudo, Mainardi, et al. 2024) may quantify the resistance and resilience capacity of a (sub‐)individual, but their relevance at the colony level needs to be demonstrated. Because the complexity and thus the level of unpredictability increase scaling up from cells to individuals and to colonies (van Straalen 2003), future studies should clarify the fine mechanisms at lower levels of biological organization that lead to colony failure and identify the tipping point, representing here the number of individuals (or cells) or functionality losses that can be tolerated before the superorganism collapses. Modeling complex interactive processes across biological levels can be useful for linking individual biomarkers to colony dynamics. However, to achieve this goal, it is important to understand how different biological levels resist and recover from stressors, and how they are able to buffer perturbations at other levels (Cavigelli et al. 2021). These mechanisms that regulate the homeostasis of (super)organisms at different levels of biological organization can be described with the cybernetic principles (e.g., self‐regulation, feedback, and network dynamics) and integrated into simulation models to enhance their ability to understand, simulate, and predict superorganism responses to environmental stressors.

## 5 A Cybernetic Perspective on Superorganisms

Cybernetics is a discipline that originally emerged to identify common principles in the functioning of automatic machines and the human nervous system. It soon expanded its scope to uncover general patterns that characterize any *complex system* involved in control and communication (Wiener 1948). Specifically, cybernetics is an important part of “systems theory” that studies systems as groups of interconnected elements organized toward a specific purpose and characterized by key features such as networks, hierarchies, and feedback loops (Stebbing 2011).

“Hierarchy” is a central concept in cybernetics and can be defined as a vertical organization of levels in complex systems, in which higher levels are dominant over lower levels that are nested within higher levels (Stebbing 2011). Each level is composed of a set of “interconnected things” (i.e., networks) that work together to reach a specific goal. From a cybernetic perspective, a complex system is a self‐regulated system characterized by negative and positive feedbacks. Negative feedbacks are reactions that tend to stabilize the system, whereas positive feedbacks are reactions that tend to amplify the effects of a perturbation. In the absence of external perturbations or stressors, the system tends to return to its *state* (e.g., a stable condition), exhibiting small or large oscillations around it. In the presence of stressors, the system can react with negative feedbacks in order to dampen the perturbation; however, in some circumstances with strong and persistent perturbations, as the system approaches a tipping point, the amplitude and duration of these oscillations typically increase till the system jumps from one *state* to another. Thus, the collapse occurs when the system loses its homeostatic capacity (Stebbing 2011).

Following a cybernetic approach, superorganisms may be viewed as complex systems characterized by emergent properties that arise from the interactions of different components that compose the system. In fact, a superorganism is organized in “nested networks” where each node (individual), at a higher level of biological organization, contains nested networks at lower levels (organs, tissues, cells) (Canciani et al. 2019). At each level, the node aggregations (e.g., specialized cells that form an organ, groups of individuals that perform the same task in the colony or groups of birds in a storm) often behave as a unit with properties that are not merely a sum of the individual behaviors. The complex set of behaviors of these aggregations, and the nested hierarchy in which life is structured (from gene to superorganism), can be explained through cybernetic principles (Wey et al. 2008; Parisi 1990; Stebbing 2011).

In this framework, it is possible to describe the dynamics of a superorganism with several positive and negative feedbacks. For example, in honeybee colonies, division of labor is age‐dependent (age polyethism) but also strongly influenced by social feedbacks. If the number of foragers declines, in‐hive bees accelerate their behavioral development and begin foraging earlier to compensate for the loss (Perry et al. 2015). Similarly, if there is a lack of nurse bees and a surplus of foragers, they can reverse their behavioral development and return from foraging to nursing roles (Seeley 1995; Robinson et al. 1992). The flexibility observable at the colony level on task allocations among workers is based on “information center strategy” where the network of worker interactions, which establishes a set of feedback mechanisms, is the result of behavioral variability and flexibility at the individual level (Seeley 1995). Similarly, the balance between the nectar and pollen foragers and the amount of sugar and protein stocks in the hive is regulated by feedback loops that have been interpreted by Schmickl and Karsai (2018) using the “common stomach” or “social crop” approach. The common stomach in social insects, like honeybees, ants, and wasps, is a shared storage system for key substances (i.e., pollen, nectar, water) that regulates task allocation. Its saturation level signals the colony's needs, allowing workers to switch tasks efficiently without complex communication or learning (Schmickl and Karsai 2018). The self‐regulation ability of the colony in the framework of the “common stomach” approach has been successfully described with a mathematical model (Schmickl and Karsai 2017). From a biological point of view, these self‐regulation mechanisms are influenced by pheromones circulating in the nest via trophallaxis, and their physiological mechanisms, which include gland maturations or regression, are well known (Winston 1987). In analogy with the organism, where the hormones regulate the functionality of different organs, the pheromones in the superorganism are responsible for the regulation of the activities among individuals. Several of these activities, including task division and colony thermoregulation, which are controlled by social feedbacks in superorganisms, have been simulated with simple mathematical models (Khoury et al. 2011; Becher et al. 2010).

Following the principle of cybernetics, a superorganism can also be conceptualized as a network in which individuals represent the nodes, and their interactions serve as the links connecting these nodes (Fewell 2003). The collapse of such a network may occur when certain nodes are lost—either in absolute numbers or as a proportion, particularly if key nodes (those with high connectivity) are affected—or when critical links disappear. This process, due to interactions with environmental stressors, can happen at different levels of biological organization, for example, at the individual level, where cells constitute the nodes and their interactions form the links. In this context, the central question regarding system collapse is to predict the threshold at which the loss of cells (nodes) or interactions (links) leads to the failure of organ functionality and, by extension, impacts the vitality of the entire organism or superorganism across scales.

Historically, scientific knowledge in ecology was mostly focused on the processes and interactions within a single level of organization (“horizontal levels”), while the study of the connections between different hierarchical levels of biological organization (“vertical links”) was only marginally considered, as it required crossing disciplinary borders (Jordán 2022). Integrating cybernetic principles and social network analysis with a multi‐level approach offers a powerful tool for linking “horizontal and vertical thinking” and thus understanding complex biological systems (Jordán 2022; Wey et al. 2008). By identifying the key nodes that interact between different levels of biological organization, we can better predict how biological systems respond to perturbations (or stressors) at various levels. This, in turn, enables us to understand how disruptions at lower organizational levels influence higher‐level functions, and vice versa (Cavigelli et al. 2021). The challenge is to deal with systems that contain a huge number of interacting components and are characterized by emergent properties that are difficult to anticipate from the knowledge of single components. For this reason, it is important to study the interactions between the components of biological systems following a holistic approach where cybernetics can play a key role (Kitano 2002). In line with this perspective, Breda et al. (2022) adopted a systems biology approach to investigate the interactions among the stress factors that affect bee health. By focusing on the properties of the colony as a system and how the stressors affect it, they found two stable equilibria that help reconcile the contrasting outcomes reported in field studies on the impacts of pesticides on honeybee colonies (Woodcock et al. 2017). Interestingly, their findings showed that the immunosuppression caused by the widespread bee pathogen, Deformed Wing Virus, can introduce a critical positive feedback loop in the system causing bistability leading to two potential final outputs in individual bees, prolonged survival or premature death, which in turn affects colony survival (Breda et al. 2022).

**FIGURE 4:**
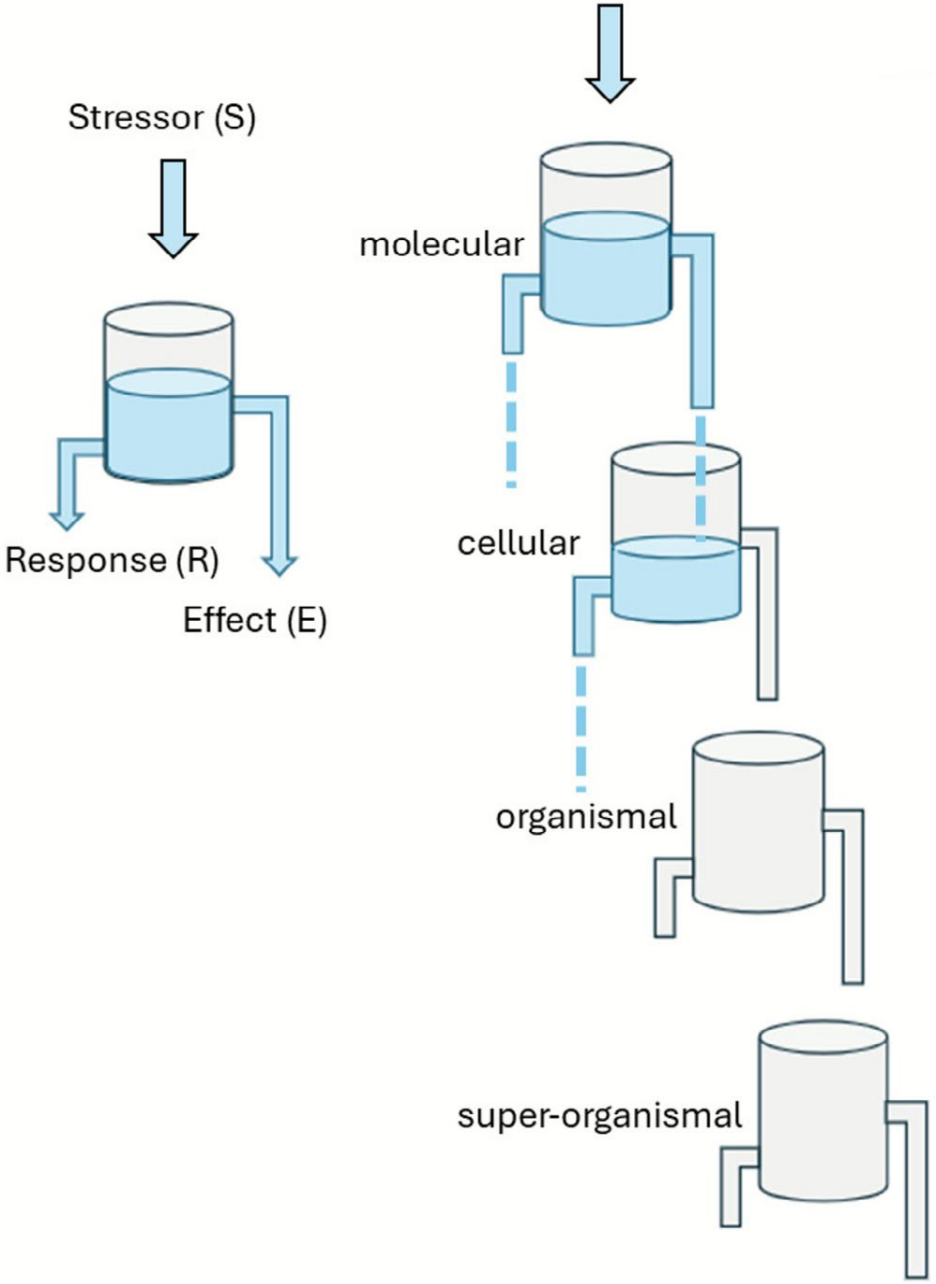
Hierarchical control is illustrated with a hydraulic model of vessels with two outlets: Inflow represents a toxic load (S), lower outflow represents the homeostatic response (R), and overflow to the following vessel produces an effect (E) visible at the next level of biological organization, illustrating the accumulation of resilience and potential cascade effects (redrawn from figure 9.7 in Stebbing 2011).

In the framework of cybernetic laws, the hierarchical system allows each level of biological organization to exert semi‐autonomous local control through feedback loops (Stebbing 2011). For instance, in insects, cells increase the production of HSPs (Heat shock proteins) in response to heat, chemical, and other stressors. However, during a stress response, the overall coordination comes from higher levels, such as the nervous and endocrine systems, which override and align local responses to ensure the whole organism reacts coherently. This hierarchical control mechanism can be illustrated with a hydraulic model consisting of a series of connected vessels, each with two outlet pipes through which water can escape (Figure 4; Stebbing 2011). The initial inflow of water into the first vessel represents, for example, a toxic load (S), while the outflow from the lower pipe corresponds to the homeostatic response (R). When the inflow rate exceeds the outflow rate (S > R), water accumulates in the vessel until it reaches the level of the second, higher outlet pipe. At this point, the overflow passes into the next vessel, producing an effect (E) that becomes apparent at the succeeding level of biological organization. Through the same mechanism, effects can propagate across successive levels (Stebbing 2011). This simple hydraulic model illustrates how resilience and resistance capacities accumulate across different levels of biological organization and how cascade effects may emerge (Figures 1 and 4).

## 6 Superorganisms as Models for Understanding Complex Systems

Superorganisms can be used as models for understanding how complex systems react to stressors and for identifying general stress response patterns and trends that apply across different levels of biological organization. The analogy between a superorganism and a Metazoan animal offers the possibility to understand how and why complex systems collapse and to address other big questions in biology. In fact, unlike other biological complex systems, such as pluricellular organisms, superorganisms can be easily experimentally manipulated to solve general problems in biology (Fewell 2003; Kennedy et al. 2017).

In the framework of the complex systems theory and cybernetic laws, the concepts of resilience, feedback loops, tipping point and emergent properties that characterize the superorganism can be extrapolated to other systems and used to understand general mechanisms of systems collapse. In a transdisciplinary way, the above‐mentioned concepts can be applied in different contexts, from the collapse of human societies to the collapse of ecological networks (Cumming and Peterson 2017), but always with the aim of understanding the mechanisms and identifying the tipping point or early warning signals that can predict whether or when collapse occurs. A similar approach has been proposed by Amdam and Seehuus (2006) to understand the molecular mechanism of cancer using the honeybee colony as a model. In the superorganism, order, which is controlled via complex signaling cascades and surveillance schemes, can be replaced by disorder, leading to collapse. This phenomenon occurs when *A. m. capensis* workers infiltrate *A. m. scutellata* colonies and trigger uncontrolled reproduction, as their eggs are accepted and their larvae are nourished like queens. The emerging adults often develop enlarged ovaries, refrain from contributing to colony tasks, and begin laying eggs, ultimately driving the colony toward collapse. This happens because the mechanisms that normally suppress worker reproduction—through pheromone‐driven inhibition of oogenesis and nest surveillance schemes enforced by the worker population—are disrupted (Amdam and Seehuus 2006). Analogously, this happens in the human body when the order is disrupted by the abnormal growth of some cells (cancerous cells) that invade other parts of the body.

The validation of the hydraulic model in Figure 4, as a hierarchical feedback‐based regulation across different biological scales, and a deeper understanding of how superorganisms react to environmental stressors can shed light on major questions in evolutionary biology, such as the origins of multicellularity or eusociality. The self‐organization and the emergent properties observed in the superorganism (high biological level) can be easily translated and applied at the individual and sub‐individual levels (lower levels), allowing a deeper understanding of the mechanisms and selective forces shaping more complex biological structures. The origin of different levels of biological organization, including organelles into cells, cells into multicellular organisms and organisms into superorganisms, can be seen as a new response in the evolution of life to maintain the stability of the system in the face of environmental stressors (Pull and McMahon 2020). However, the specific factors driving the evolutionary transition toward new and more complex forms of biological organization remain underexplored. The cybernetic and multilevel approach proposed in this paper to understand stress responses in superorganisms can provide insight into how and why new complex biological structures arose on our planet.

## 7 Conclusions

Superorganisms have evolved an additional level of organismality that enables them to better withstand environmental stressors than individual organisms. However, under certain conditions, often difficult to predict, superorganisms can rapidly collapse. The key challenge lies in understanding the underlying mechanisms and causes of such collapses.

Predicting when and why the collapse of the superorganism occurs is fundamental for pest control against superorganisms like ant and termite colonies (Box 1) or proposing mitigation strategies for protecting useful superorganisms for human wellbeing like honeybee colonies (More et al. 2021).

While numerous laboratory studies have demonstrated the effects of stressors at lower levels of biological organization (e.g., molecular, cellular, and tissue levels), the ecological significance of these effects at higher levels remains largely underexplored. Moreover, it is not well understood how impacts observed at lower levels scale up to influence higher organizational levels, nor which endpoints (i.e., measurable biological responses) might serve as reliable early warning indicators of critical tipping points. Addressing these knowledge gaps necessitates an integrated approach that combines experimental and computational investigations and links “horizontal and vertical thinking” (Jordán 2022). This innovative operative approach aims to apply the cybernetic law and the network analysis to understand stress response with a special focus on vertical links connecting organizational levels.

Given the complexity of interactions between stressors and superorganisms, modeling approaches are indispensable for identifying key factors and predicting the fundamental patterns that precede superorganism collapse. Within this context, cybernetics offers a powerful framework for analyzing systems where direct observation of all components is not feasible. According to the cybernetic perspective, collapse can be interpreted as the system's failure to maintain homeostasis (Figure 4) (Stebbing 2011). This conceptual framework can be applicable across multiple levels of biological organization characteristic of superorganisms and, more broadly, provides a robust basis for explaining stress responses in complex systems and sheds light on fundamental biological questions, such as the evolution of eusociality and the origins of multicellularity.

## Author Contributions

**Fabio Sgolastra:** conceptualization (lead), investigation (lead), project administration (lead), writing – original draft (lead), writing – review and editing (lead).

## Funding

This work was funded by the European Union within the framework of the Next Generation EU ‐ PNRR.

## Conflicts of Interest

The author declares no conflicts of interest.

## Data Availability

The author has nothing to report.

## References

[ece372626-bib-0001] Amdam, G. V. , and S. C. Seehuus . 2006. “Order, Disorder, Death: Lessons From a Superorganism.” Advances in Cancer Research 95: 31–60. 10.1016/S0065-230X(06)95002-7. 16860655 PMC2386248

[ece372626-bib-0002] Baggio, J. A. , K. Brown , and D. Hellebrandt . 2015. “Boundary Object or Bridging Concept? A Citation Network Analysis of Resilience.” Ecology and Society 20, no. 2: art2. 10.5751/ES-07484-200202.

[ece372626-bib-0003] Barron, A. B. 2015. “Death of the Bee Hive: Understanding the Failure of an Insect Society.” Current Opinion in Insect Science 10: 45–50. 10.1016/j.cois.2015.04.004. 29588013

[ece372626-bib-0004] Bastiaansen, R. , A. Doelman , F. Van Langevelde , and V. Rottschafer . 2020. “Modeling Honey Bee Colonies in Winter Using a Keller‐Segel Model With a Sign‐Changing Chemotactic Coefficient.” SIAM Journal on Applied Mathematics 80, no. 2: 839–863. 10.1137/19M1246067.

[ece372626-bib-0005] Becher, M. A. , H. Hildenbrandt , C. K. Hemelrijk , and R. F. A. Moritz . 2010. “Brood Temperature, Task Division and Colony Survival in Honeybees: A Model.” Ecological Modelling 221, no. 5: 769–776. 10.1016/j.ecolmodel.2009.11.016.

[ece372626-bib-0006] Berenbaum, M. R. , and L. H. Liao . 2019. “Honey Bees and Environmental Stress: Toxicologic Pathology of a Superorganism.” Toxicologic Pathology 47, no. 8: 1076–1081. 10.1177/0192623319877154. 31581932

[ece372626-bib-0007] Bordoni, A. , M. A. Miroddi , L. Dapporto , and S. Turillazzi . 2017. “Long‐Term Assessment Reveals the Hidden and Hiding Effects of Experimental Stress on Ant Colonies.” Behavioral Ecology and Sociobiology 71, no. 10: 144. 10.1007/s00265-017-2373-6.

[ece372626-bib-0008] Breda, D. , D. Frizzera , G. Giordano , et al. 2022. “A Deeper Understanding of System Interactions Can Explain Contradictory Field Results on Pesticide Impact on Honey Bees.” Nature Communications 13, no. 1: 5720. 10.1038/s41467-022-33405-7. PMC9523045 36175425

[ece372626-bib-0009] Bryden, J. , R. J. Gill , R. A. A. Mitton , N. E. Raine , and V. A. A. Jansen . 2013. “Chronic Sublethal Stress Causes Bee Colony Failure.” Ecology Letters 16: 1463–1469. 10.1111/ele.12188. 24112478 PMC4299506

[ece372626-bib-0010] Calabrese, E. J. , and L. A. Baldwin . 2001. “U‐Shaped Dose‐Responses in Biology, Toxicology, and Public Health.” Annual Review of Public Health 22, no. 1: 15–33. 10.1146/annurev.publhealth.22.1.15. 11274508

[ece372626-bib-0011] Caliani, I. , T. Campani , B. Conti , et al. 2021. “Multi‐Biomarker Approach and IBR Index to Evaluate the Effects of Different Contaminants on the Ecotoxicological Status of *Apis mellifera* .” Ecotoxicology and Environmental Safety 208: 111486. 10.1016/j.ecoenv.2020.111486. 33130481

[ece372626-bib-0012] Canciani, M. , A. Arnellos , and A. Moreno . 2019. “Revising the Superorganism: An Organizational Approach to Complex Eusociality.” Frontiers in Psychology 10: 2653. 10.3389/fpsyg.2019.02653. 31849768 PMC6901679

[ece372626-bib-0013] Cavigelli, S. , J. Leips , Q. Y. Xiang , Jenny , D. Lemke , and N. Konow . 2021. “Next Steps in Integrative Biology: Mapping Interactive Processes Across Levels of Biological Organization.” Integrative and Comparative Biology 61, no. 6: 2066–2074. 10.1093/icb/icab161. 34259855

[ece372626-bib-0014] Chen, J. , Y.‐J. Liu , Q. Wang , et al. 2024. “Multiple Stresses Induced by Chronic Exposure to Flupyradifurone Affect Honey Bee Physiological States.” Science of the Total Environment 935: 173418. 10.1016/j.scitotenv.2024.173418. 38788938

[ece372626-bib-0015] Chen, X. , A. Li , L. Yin , L. Ke , P. Dai , and Y.‐J. Liu . 2023. “Early‐Life Sublethal Thiacloprid Exposure to Honey Bee Larvae: Enduring Effects on Adult Bee Cognitive Abilities.” Toxics 12, no. 1: 18. 10.3390/toxics12010018. 38250974 PMC10820931

[ece372626-bib-0016] Colin, T. , V. Dakos , A. Barron , W. Meikle , E. Altmann , and T. Latty . 2024. Early Warnings of Bee Colony Failure. Research Square (Preprint version 1). 10.21203/rs.3.rs-3712390/v1.

[ece372626-bib-0017] Crall, J. D. , and N. E. Raine . 2023. “How Do Neonicotinoids Affect Social Bees? Linking Proximate Mechanisms to Ecological Impacts.” Advances in Insect Physiology 64: 191–253. 10.1016/bs.aiip.2023.01.004.

[ece372626-bib-0018] Cremer, S. , S. A. O. Armitage , and P. Schmid‐Hempel . 2007. “Social Immunity.” Current Biology 17, no. 16: R693–R702. 10.1016/j.cub.2007.06.008. 17714663

[ece372626-bib-0019] Cremer, S. , and C. D. Pull . 2024. “Unconditional Versus Condition‐Dependent Social Immunity.” Trends in Parasitology 40, no. 9: 780–787. 10.1016/j.pt.2024.07.014. 39152078

[ece372626-bib-0020] Cresswell, J. E. 2011. “A Meta‐Analysis of Experiments Testing the Effects of a Neonicotinoid Insecticide (Imidacloprid) on Honey Bees.” Ecotoxicology 20, no. 1: 149–157. 10.1007/s10646-010-0566-0. 21080222

[ece372626-bib-0021] Cumming, G. S. , and G. D. Peterson . 2017. “Unifying Research on Social–Ecological Resilience and Collapse.” Trends in Ecology & Evolution 32, no. 9: 695–713. 10.1016/j.tree.2017.06.014. 28734593

[ece372626-bib-0022] Dennis, B. , and W. P. Kemp . 2016. “How Hives Collapse: Allee Effects, Ecological Resilience, and the Honey Bee.” PLoS One 11, no. 2: e0150055. 10.1371/journal.pone.0150055. 26910061 PMC4765896

[ece372626-bib-0023] Desneux, N. , A. Decourtye , and J. M. Delpuech . 2007. “The Sublethal Effects of Pesticides on Beneficial Arthropods.” Annual Review of Entomology 52: 81–106. 10.1146/annurev.ento.52.110405.091440. 16842032

[ece372626-bib-0024] Di Prisco, G. , V. Cavaliere , D. Annoscia , et al. 2013. “Neonicotinoid Clothianidin Adversely Affects Insect Immunity and Promotes Replication of a Viral Pathogen in Honey Bees.” Proceedings of the National Academy of Sciences of the United States of America 110, no. 46: 18466–18471. 10.1073/pnas.1314923110. 24145453 PMC3831983

[ece372626-bib-0025] Doublet, V. , M. Labarussias , J. R. de Miranda , R. F. A. Moritz , and R. J. Paxton . 2014. “Bees Under Stress: Sublethal Doses of a Neonicotinoid Pesticide and Pathogens Interact to Elevate Honey Bee Mortality Across the Life Cycle.” Environmental Microbiology 17: 969–983. 10.1111/1462-2920.12426. 25611325

[ece372626-bib-0026] Easton‐Calabria, A. C. , J. A. Thuma , K. Cronin , et al. 2023. “Colony Size Buffers Interactions Between Neonicotinoid Exposure and Cold Stress in Bumblebees.” Proceedings of the Royal Society B: Biological Sciences 290, no. 2003: 20230555. 10.1098/rspb.2023.0555. PMC10354472 37464757

[ece372626-bib-0027] EFSA (European Food Safety Authority) . 2016. “A Mechanistic Model to Assess Risks to Honeybee Colonies From Exposure to Pesticides Under Different Scenarios of Combined Stressors and Factors.” Technical Report 13, no. 7: 1069E. 10.2903/sp.efsa.2016.4653.

[ece372626-bib-0028] Elizabeth, M. , and H. Robert . 2015. A Dictionary of Biology. 7th ed. Oxford University Press.

[ece372626-bib-0029] Fewell, J. H. 2003. “Social Insect Networks.” Science 301: 1867–1870. 14512616 10.1126/science.1088945

[ece372626-bib-0030] Gandra, L. C. , K. D. Amaral , J. C. Couceiro , T. M. Della Lucia , and R. N. Guedes . 2016. “Mechanism of Leaf‐Cutting Ant Colony Suppression by Fipronil Used in Attractive Toxic Baits.” Pest Management Science 72, no. 8: 1475–1481. 10.1002/ps.4239. 26817422

[ece372626-bib-0031] Groeneveld, J. , R. Odemer , and F. Requier . 2024. “Brood Indicators Are an Early Warning Signal of Honey Bee Colony Loss—A Simulation‐Based Study.” PLoS One 19: e0302907. 10.1371/journal.pone.0302907. 38753826 PMC11098398

[ece372626-bib-0032] Henry, M. , N. Cerrutti , P. Aupinel , et al. 2015. “Reconciling Laboratory and Field Assessments of Neonicotinoid Toxicity to Honeybees.” Proceedings of the Royal Society B: Biological Sciences 282, no. 1819: 2110. 10.1098/rspb.2015.2110. PMC4685821 26582026

[ece372626-bib-0033] Jordán, F. 2022. “The Network Perspective: Vertical Connections Linking Organizational Levels.” In Ecological Modelling, vol. 473, 110112. Elsevier B.V. 10.1016/j.ecolmodel.2022.110112.

[ece372626-bib-0034] Kennedy, P. , G. Baron , B. Qiu , et al. 2017. “Deconstructing Superorganisms and Societies to Address Big Questions in Biology.” Trends in Ecology & Evolution 32, no. 11: 861–872. 10.1016/j.tree.2017.08.004. 28899581

[ece372626-bib-0035] Khoury, D. S. , M. R. Myerscough , and A. B. Barron . 2011. “A Quantitative Model of Honey Bee Colony Population Dynamics.” PLoS One 6, no. 4: e18491. 10.1371/journal.pone.0018491. 21533156 PMC3078911

[ece372626-bib-0036] Kitano, H. 2002. “Computational Systems Biology.” Nature 420, no. 6912: 206–210. 10.1038/nature01254. 12432404

[ece372626-bib-0037] Klein, S. , A. Cabirol , J. M. Devaud , A. B. Barron , and M. Lihoreau . 2017. “Why Bees Are So Vulnerable to Environmental Stressors.” Trends in Ecology & Evolution 32, no. 4: 268–278. 10.1016/j.tree.2016.12.009. 28111032

[ece372626-bib-0038] Kohl, P. , E. J. Crampin , T. A. Quinn , and D. Noble . 2010. “Systems Biology: An Approach.” Clinical Pharmacology & Therapeutics 88, no. 1: 25–33. 10.1038/clpt.2010.92. 20531468

[ece372626-bib-0039] Koto, A. , M. Tamura , P. S. Wong , et al. 2023. “Social Isolation Shortens Lifespan Through Oxidative Stress in Ants.” Nature Communications 14, no. 1: 5493. 10.1038/s41467-023-41140-w. PMC10533837 37758727

[ece372626-bib-0040] Kültz, D. 2005. “Molecular and Evolutionary Basis of the Cellular Stress Response.” Annual Review of Physiology 67: 225–257. 10.1146/annurev.physiol.67.040403.103635 15709958

[ece372626-bib-0041] Latty, T. , and V. Dakos . 2019. “The Risk of Threshold Responses, Tipping Points, and Cascading Failures in Pollination Systems.” Biodiversity and Conservation 28, no. 13: 3389–3406. 10.1007/s10531-019-01844-2.

[ece372626-bib-0042] Lu, C. , C. H. Chang , B. Lemos , Q. Zhang , and D. MacIntosh . 2020. “Mitochondrial Dysfunction: A Plausible Pathway for Honeybee Colony Collapse Disorder (CCD).” Environmental Science and Technology Letters 7, no. 4: 254–258. 10.1021/acs.estlett.0c00070.

[ece372626-bib-0043] Lu, C. , K. M. Warchol , and R. A. Callahan . 2012. “In Situ Replication of Honey Bee Colony Collapse Disorder.” Bulletin of Insectology 65, no. 1: 99–106.

[ece372626-bib-0044] Medrzycki, P. , F. Sgolastra , L. Bortolotti , et al. 2010. “Influence of Brood Rearing Temperature on Honey Bee Development and Susceptibility to Poisoning by Pesticides.” Journal of Apicultural Research 49, no. 1: 52–59. 10.3896/IBRA.1.49.1.07.

[ece372626-bib-0045] Miller, B. F. , D. R. Seals , and K. L. Hamilton . 2017. “A Viewpoint on Considering Physiological Principles to Study Stress Resistance and Resilience With Aging.” Ageing Research Reviews 38: 1–5. 10.1016/j.arr.2017.06.004. 28676286

[ece372626-bib-0046] Minaud, É. , F. Rebaudo , P. Davidson , et al. 2024. “How Stressors Disrupt Honey Bee Biological Traits and Overwintering Mechanisms.” Heliyon 10, no. 14: e34390. 10.1016/j.heliyon.2024.e34390. 39108870 PMC11301357

[ece372626-bib-0047] Minaud, E. , F. Rebaudo , G. Mainardi , et al. 2024. “Temperature in Overwintering Honey Bee Colonies Reveals Brood Status and Predicts Colony Mortality.” Ecological Indicators 169: 112961. 10.1016/j.ecolind.2024.112961.

[ece372626-bib-0048] Miotelo, L. , A. L. Mendes dos Reis , A. Rosa‐Fontana , et al. 2022. “A Food‐Ingested Sublethal Concentration of Thiamethoxam Has Harmful Effects on the Stingless Bee *Melipona scutellaris* .” Chemosphere 288: 132461. 10.1016/j.chemosphere.2021.132461. 34624342

[ece372626-bib-0049] More, S. , V. Bampidis , D. Benford , et al. 2021. “A Systems‐Based Approach to the Environmental Risk Assessment of Multiple Stressors in Honey Bees.” EFSA Journal 19: 6607. 10.2903/j.efsa.2021.6607. PMC8135085 34025804

[ece372626-bib-0050] Nowak, M. A. , C. E. Tarnita , and E. O. Wilson . 2010. “The Evolution of Eusociality.” Nature 466, no. 7310: 1057–1062. 10.1038/nature09205. 20740005 PMC3279739

[ece372626-bib-0051] Oldroyd, B. P. 2007. “What's Killing American Honey Bees?” PLoS Biology 5, no. 6: e168. 10.1371/journal.pbio.0050168. 17564497 PMC1892840

[ece372626-bib-0052] Orr, J. A. , R. D. Vinebrooke , M. C. Jackson , et al. 2020. “Towards a Unified Study of Multiple Stressors: Divisions and Common Goals Across Research Disciplines.” Proceedings of the Royal Society B: Biological Sciences 287, no. 1926: 20200421. 10.1098/rspb.2020.0421. PMC7282922 32370677

[ece372626-bib-0053] Parisi, G. 1990. “A Simple Model for the Immune Network.” Proceedings of the National Academy of Sciences of the United States of America 87: 429–433. https://www.pnas.org. 2296597 10.1073/pnas.87.1.429 PMC53277

[ece372626-bib-0054] Parr, C. L. , and T. R. Bishop . 2022. “The Response of Ants to Climate Change.” Global Change Biology 28, no. 10: 3188–3205. 10.1111/gcb.16140. 35274797 PMC9314018

[ece372626-bib-0055] Perry, C. J. , E. Søvik , M. R. Myerscough , and A. B. Barron . 2015. “Rapid Behavioral Maturation Accelerates Failure of Stressed Honey Bee Colonies.” Proceedings of the National Academy of Sciences of the United States of America 112, no. 11: 3427–3432. 10.1073/pnas.1422089112. 25675508 PMC4371971

[ece372626-bib-0056] Pohl, M. , M. Otto , U. Hommen , et al. 2025. “A Staged Approach Using Ants as Test Organism in Ecotoxicology.” Science of The Total Environment 983: 179669. 10.1101/2025.03.04.641392. 40398167

[ece372626-bib-0057] Pull, C. D. , and D. P. McMahon . 2020. “Superorganism Immunity: A Major Transition in Immune System Evolution.” Frontiers in Ecology and Evolution 8: 186. 10.3389/fevo.2020.00186.

[ece372626-bib-0058] Rafael Braga, A. , D. G. Gomes , R. Rogers , E. E. Hassler , B. M. Freitas , and J. A. Cazier . 2020. “A Method for Mining Combined Data From In‐Hive Sensors, Weather and Apiary Inspections to Forecast the Health Status of Honey Bee Colonies.” Computers and Electronics in Agriculture 169: 105161. 10.1016/j.compag.2019.105161.

[ece372626-bib-0059] Robinson, G. E. , R. E. Page , C. Strambi , and A. Strambi . 1992. “Colony Integration in Honey Bees: Mechanisms of Behavioral Reversion.” Ethology 90, no. 4: 336–348. 10.1111/j.1439-0310.1992.tb00844.x.

[ece372626-bib-0060] Rosenberg, Y. , Y. M. Bar‐On , A. Fromm , et al. 2023. “ECOLOGY the Global Biomass and Number of Terrestrial Arthropods.” 10.1126/sciadv.abq4049 PMC9897674 36735788

[ece372626-bib-0061] Santos, A. V. , B. L. De Oliveira , and R. I. Samuels . 2007. “Selection of Entomopathogenic Fungi for Use in Combination With Sub‐Lethal Doses of Imidacloprid: Perspectives for the Control of the Leaf‐Cutting Ant *Atta sexdens* Rubropilosa Forel (Hymenoptera: Formicidae).” Mycopathologia 163, no. 4: 233–240. 10.1007/s11046-007-9009-8. 17404893

[ece372626-bib-0062] Scharf, I. , A. P. Modlmeier , S. Beros , and S. Foitzik . 2012. “Ant Societies Buffer Individual‐Level Effects of Parasite Infections.” American Naturalist 180, no. 5: 671–683. 10.1086/667894. 23070326

[ece372626-bib-0063] Scheffer, M. , J. E. Bolhuis , D. Borsboom , et al. 2018. “Quantifying Resilience of Humans and Other Animals.” Proceedings of the National Academy of Sciences 115, no. 47: 11883–11890. 10.1073/pnas.1810630115. PMC6255191 30373844

[ece372626-bib-0064] Schläppi, D. , N. Kettler , G. Glauser , L. Straub , O. Yañez , and P. Neumann . 2021. “Varying Impact of Neonicotinoid Insecticide and Acute Bee Paralysis Virus Across Castes and Colonies of Black Garden Ants, *Lasius Niger* (Hymenoptera: Formicidae).” Scientific Reports 11, no. 1: 20500. 10.1038/s41598-021-98406-w. 34654848 PMC8519937

[ece372626-bib-0065] Schmickl, T. , and I. Karsai . 2017. “Resilience of Honeybee Colonies via Common Stomach: A Model of Self‐Regulation of Foraging.” PLoS One 12, no. 11: e0188004. 10.1371/journal.pone.0188004. 29161278 PMC5697885

[ece372626-bib-0066] Schmickl, T. , and I. Karsai . 2018. “Integral Feedback Control Is at the Core of Task Allocation and Resilience of Insect Societies.” Proceedings of the National Academy of Sciences of the United States of America 115, no. 52: 13180–13185. 10.1073/pnas.1807684115. 30530662 PMC6310805

[ece372626-bib-0067] Schott, M. , M. Sandmann , J. E. Cresswell , et al. 2021. “Honeybee Colonies Compensate for Pesticide‐Induced Effects on Royal Jelly Composition and Brood Survival With Increased Brood Production.” Scientific Reports 11, no. 1: 62. 10.1038/s41598-020-79660-w. 33420177 PMC7794607

[ece372626-bib-0068] Seeley, T. D. 1995. The Wisdom of the Hive. The Social Physiology of Honey Bee Colonies. Harvard University Press.

[ece372626-bib-0069] Selye, H. 1956. The Stress of Life. McGraw‐Hill Book Company.

[ece372626-bib-0070] Sgolastra, F. , P. Medrzycki , L. Bortolotti , et al. 2020. “Bees and Pesticide Regulation: Lessons From the Neonicotinoid Experience.” Biological Conservation 241: 108356. 10.1016/j.biocon.2019.108356.

[ece372626-bib-0071] Starr, C. K. 2021. “Eusociality.” In Encyclopedia of Social Insects, edited by C. K. Starr . Springer.

[ece372626-bib-0072] Stebbing, T. 2011. A Cyernetic View of Biological Growth: The Maja Hypothesis. Cambridge University Press.

[ece372626-bib-0073] Steinberg, C. E. W. 2012. Stress Ecology. Springer Science+Business Media B.V.

[ece372626-bib-0074] Straub, L. , G. R. Williams , J. Pettis , I. Fries , and P. Neumann . 2015. “Superorganism Resilience: Eusociality and Susceptibility of Ecosystem Service Providing Insects to Stressors.” Current Opinion in Insect Science 12: 109–112. 10.1016/j.cois.2015.10.010.

[ece372626-bib-0075] Sulmon, C. , J. Van Baaren , F. Cabello‐Hurtado , et al. 2015. “Abiotic Stressors and Stress Responses: What Commonalities Appear Between Species Across Biological Organization Levels?” Environmental Pollution 202: 66–77. 10.1016/j.envpol.2015.03.013. 25813422

[ece372626-bib-0076] Svoboda, J. , P. Pech , and P. Heneberg . 2023. “Low Concentrations of Acetamiprid, Deltamethrin, and Sulfoxaflor, Three Commonly Used Insecticides, Adversely Affect Ant Queen Survival and Egg Laying.” Scientific Reports 13, no. 1: 14893. 10.1038/s41598-023-42129-7. 37689830 PMC10492783

[ece372626-bib-0077] Tadei, R. , G. Cilia , E. C. Mathias Da Silva , et al. 2025. “Co‐Exposure to a Honeybee Pathogen and an Insecticide: Synergistic Effects in a New Solitary Bee Host but Not in *Apis mellifera* .” Proceedings of the Royal Society B: Biological Sciences 292, no. 2042: 20242809. 10.1098/rspb.2024.2809. PMC11881019 40041960

[ece372626-bib-0078] Tosi, S. , C. Sfeir , E. Carnesecchi , D. vanEngelsdorp , and M. P. Chauzat . 2022. “Lethal, Sublethal, and Combined Effects of Pesticides on Bees: A Meta‐Analysis and New Risk Assessment Tools.” Science of the Total Environment 844: 156857. 10.1016/j.scitotenv.2022.156857. 35760183

[ece372626-bib-0079] Ulgezen, Z. N. , C. van Dooremalen , and F. Langevelde . 2021. “Understanding Social Resilience in Honeybee Colonies.” Current Research in Insect Science 1: 100021. 10.1016/j.cris.2021.100021. 36003609 PMC9387495

[ece372626-bib-0080] Van Meerbeek, K. , T. Jucker , and J. C. Svenning . 2021. “Unifying the Concepts of Stability and Resilience in Ecology.” Journal of Ecology 109, no. 9: 3114–3132. 10.1111/1365-2745.13651.

[ece372626-bib-0081] van Straalen, N. M. 2003. “Straalen‐2003‐Stress‐Ecology.” Environmental Science & Technology 37: 324A–330A. 10.1021/es0325720 12967088

[ece372626-bib-0082] vanEngelsdorp, D. , J. D. Evans , C. Saegerman , et al. 2009. “Colony Collapse Disorder: A Descriptive Study.” PLoS One 4, no. 8: e6481. 10.1371/journal.pone.0006481. 19649264 PMC2715894

[ece372626-bib-0083] Vanengelsdorp, D. , and M. D. Meixner . 2010. “A Historical Review of Managed Honey Bee Populations in Europe and the United States and the Factors That May Affect Them.” Journal of Invertebrate Pathology 103, no. 2010: S80–S95. 10.1016/j.jip.2009.06.011. 19909973

[ece372626-bib-0084] Walton, A. , J. J. Herman , and O. Rueppell . 2024. “Social Life Results in Social Stress Protection: A Novel Concept to Explain Individual Life‐History Patterns in Social Insects.” Biological Reviews 99, no. 4: 1444–1457. 10.1111/brv.13074. 38468146

[ece372626-bib-0085] Wessler, I. , H. A. Gärtner , R. Michel‐Schmidt , et al. 2016. “Honeybees Produce Millimolar Concentrations of Non‐Neuronal Acetylcholine for Breeding: Possible Adverse Effects of Neonicotinoids.” PLoS One 11, no. 6: e0156886. 10.1371/journal.pone.0156886. 27285384 PMC4902251

[ece372626-bib-0086] Wey, T. , D. T. Blumstein , W. Shen , and F. Jordán . 2008. “Social Network Analysis of Animal Behaviour: A Promising Tool for the Study of Sociality.” Animal Behaviour 75, no. 2: 333–344. 10.1016/j.anbehav.2007.06.020.

[ece372626-bib-0087] Wheeler, W. M. 1910. The Ants: Their Structure, Development and Behaviour. Columbia University Press.

[ece372626-bib-0088] Wheeler, W. M. 1911. “The Ant‐Colony as an Organism.” Journal of Morphology 22: 307–325.

[ece372626-bib-0089] Wheeler, W. M. 1928. The Social Insects, Their Origin and Evolution. Harcourt Brace.

[ece372626-bib-0090] Wiener, N. 1948. “Cybernetics.” Scientific American 179, no. 5: 14–19. 10.2307/24945913. 18890151

[ece372626-bib-0091] Wilson, E. O. 1990. Success and Dominance in Ecosystems:The Case of the Social Insects. International Ecology Institute.

[ece372626-bib-0092] Wilson, E. O. , and B. Hölldobler . 2005. “Eusociality: Origin and Consequences.” Proceedings of the National Academy of Sciences of the United States of America 102, no. 102: 13367–13371. 10.1073/pnas.0505858102. 16157878 PMC1224642

[ece372626-bib-0093] Winston, M. L. 1987. The Biology of the Honey Bee. Harvard University Press.

[ece372626-bib-0094] Woodcock, B. A. , J. M. Bullock , R. F. Shore , et al. 2017. “Country‐Specific Effects of Neonicotinoid Pesticides on Honey Bees and Wild Bees.” Science 356: 1393–1395. 28663502 10.1126/science.aaa1190

[ece372626-bib-0095] Woodcock, B. A. , M. S. Heard , M. S. Jitlal , et al. 2016. “Replication, Effect Sizes and Identifying the Biological Impacts of Pesticides on Bees Under Field Conditions.” Journal of Applied Ecology 53, no. 5: 1358–1362. 10.1111/1365-2664.12676.

